# Supplementary material for: The GTPase activating protein Gyp7 regulates Rab7/Ypt7 activity on late endosomes
Source: J Cell Biol. 2024 Mar 27;223(6):e202305038. doi: 10.1083/jcb.202305038 (PMC10978497; doi:10.1083/jcb.202305038)
Supplement: Table S1 — shows strains used in this study. [file JCB_202305038_TableS1.docx]

**Table S1 Strains used in this study**

| **Strain** | **Genotype** | **Reference** |
| --- | --- | --- |
| SEY6210 | MATα *leu2-3,112 ura3-52 his3-∆200 trp-∆901 lys2-801 suc2-∆9 GAL* | Reggiori Laboratory |
| BY4732 | MATa *his3∆200 leu2∆0 met15∆0 trp1∆63 ura3∆0* | Boeke laboratory |
| CUY12832 | MATα *leu2-3,112 ura3-52 his3-∆200 trp-∆901 lys2-801 suc2-∆9 GAL GYP7::mNeon-natNT2* | This study |
| CUY12882 | MATα *leu2-3,112 ura3-52 his3-∆200 trp-∆901 lys2-801 suc2-∆9 GAL vps4::URA3 GYP7::mNeon-natNT2* | This study |
| CUY13729 | MATα *leu2-3,112 ura3-52 his3-∆200 trp-∆901 lys2-801 suc2-∆9 GAL vps4::URA3 GYP7::mNeon-natNT2 VPS35::2xmKate-hphNT1* | This study |
| CUY13730 | MATα *leu2-3,112 ura3-52 his3-∆200 trp-∆901 lys2-801 suc2-∆9 GAL vps4::URA3 GYP7::mNeon-natNT2 VPS21::hphNT1-PHO5pr-mCherry* | This study |
| CUY13135 | MATα *leu2-3,112 ura3-52 his3-∆200 trp-∆901 lys2-801 suc2-∆9 GAL ypt52::hphNT1 GYP7::mNeon-natNT2* | This study |
| CUY13136 | MATα *leu2-3,112 ura3-52 his3-∆200 trp-∆901 lys2-801 suc2-∆9 GAL ypt10::hphNT1*  *GYP7::mNeon-natNT2* | This study |
| CUY13206 | MATα *leu2-3,112 ura3-52 his3-∆200 trp-∆901 lys2-801 suc2-∆9 GAL vps21::trp GYP7::mNeon-natNT2* | This study |
| CUY13207 | MATα *leu2-3,112 ura3-52 his3-∆200 trp-∆901 lys2-801 suc2-∆9 GAL ypt53::hphNT1 GYP7::mNeon-natNT2* | This study |
| CUY13345 | MATα *leu2-3,112 ura3-52 his3-∆200 trp-∆901 lys2-801 suc2-∆9 GAL GYP7::mNeon-natNT2 vps9::hphNT1 muk1::kanMX* | This study |
| CUY13346 | MATα *leu2-3,112 ura3-52 his3-∆200 trp-∆901 lys2-801 suc2-∆9 GAL vps21::Trp GYP7::mNeon-natNT2 ypt52::hphNT1* | This study |
| CUY12833 | MATα *leu2-3,112 ura3-52 his3-∆200 trp-∆901 lys2-801 suc2-∆9 GAL MSB3::mNeon-natNT2* | This study |
| CUY12883 | MATα *leu2-3,112 ura3-52 his3-∆200 trp-∆901 lys2-801 suc2-∆9 GAL vps4::URA3 MSB3::mNeon-natNT2* | This study |
| CUY13520 | MATα *leu2-3,112 ura3-52 his3-∆200 trp-∆901 lys2-801 suc2-∆9 GAL ATG8::ADHpr-mCherry-natNT2* | Ungermann laboratory |
| CUY13955 | MATα *leu2-3,112 ura3-52 his3-∆200 trp-∆901 lys2-801 suc2-∆9 GAL ATG8::ADHpr-mCherry-natNT2 gyp7::hphNT1* | This study |
| CUY13956 | MATα *leu2-3,112 ura3-52 his3-∆200 trp-∆901 lys2-801 suc2-∆9 GAL ATG8::ADHpr-mCherry-natNT2 GYP7::kanMX-TEF1pr* | This study |
| CUY14115 | MATα *leu2-3,112 ura3-52 his3-∆200 trp-∆901 lys2-801 suc2-∆9 GAL GYP7::mNeon-natNT2 VPS39::hphNT1-TEF1pr-mCherry* | This study |
| CUY14222 | MATα *leu2-3,112 ura3-52 his3-∆200 trp-∆901 lys2-801 suc2-∆9 GAL GYP7::URA-TEF1pr VPS39::hphNT1-TEF1pr-mCherry* | This study |
| CUY14223 | MATα *leu2-3,112 ura3-52 his3-∆200 trp-∆901 lys2-801 suc2-∆9 GAL gyp7::natNT2 VPS39::hphNT1-TEF1pr-mCherry* | This study |
| CUY14265 | MATα *leu2-3,112 ura3-52 his3-∆200 trp-∆901 lys2-801 suc2-∆9 GAL GYP7::mNeon-natNT2 vps45::kanMX* | This study |
| CUY14266 | MATα *leu2-3,112 ura3-52 his3-∆200 trp-∆901 lys2-801 suc2-∆9 GAL GYP7::mNeon-natNT2 vps3::kanMX* | This study |
| CUY13220 | MATα *leu2-3,112 ura3-52 his3-∆200 trp-∆901 lys2-801 suc2-∆9 GAL GYP7::mNeon-natNT2 vps35::hphNT1* | This study |
| CUY13253 | MATα *leu2-3,112 ura3-52 his3-∆200 trp-∆901 lys2-801 suc2-∆9 GAL GYP7::mNeon-natNT2 vps5::hphNT1* | This study |
| CUY13262 | MATα *leu2-3,112 ura3-52 his3-∆200 trp-∆901 lys2-801 suc2-∆9 GAL GYP7::mNeon-natNT2 mvp1::hphNT1* | This study |
| CUY13263 | MATα *leu2-3,112 ura3-52 his3-∆200 trp-∆901 lys2-801 suc2-∆9 GAL GYP7::mNeon-natNT2 snx4::hphNT1* | This study |
| CUY13335 | MATα *leu2-3,112 ura3-52 his3-∆200 trp-∆901 lys2-801 suc2-∆9 GAL GYP7::mNeon-natNT2 vps38::kanMX* | This study |
| CUY12521 | MATα *leu2-3,112 ura3-52 his3-∆200 trp-∆901 lys2-801 suc2-∆9 GAL vps21::trp* | Langemeyer et al., 2020 |
| CUY8197 | MATα *leu2-3,112 ura3-52 his3-∆200 trp-∆901 lys2-801 suc2-∆9 GAL gyp7::natNT2* | John Peter et al., 2013 |
| CUY8262 | MATα *leu2-3,112 ura3-52 his3-∆200 trp-∆901 lys2-801 suc2-∆9 GAL msb3::natNT2* | John Peter et al., 2013 |
| CUY12962 | MATα *leu2-3,112 ura3-52 his3-∆200 trp-∆901 lys2-801 suc2-∆9 GAL GYP7::GFP-hphNT1* | This study |
| CUY13902 | MATα *leu2-3,112 ura3-52 his3-∆200 trp-∆901 lys2-801 suc2-∆9 GAL gyp7::natNT2 LEU2::pRS405-Gyp7pr-Gyp7-mNeon* | This study |
| CUY13391 | MATα *leu2-3,112 ura3-52 his3-∆200 trp-∆901 lys2-801 suc2-∆9 GAL gyp7::natNT2 MUP1::GFP-hphNT1* | This study |
| CUY12940 | MATα *leu2-3,112 ura3-52 his3-∆200 trp-∆901 lys2-801 suc2-∆9 GAL MUP1::GFP-hphNT1* | Ungermann laboratory |
| CUY11987 | MATα *leu2-3,112 ura3-52 his3-∆200 trp-∆901 lys2-801 suc2-∆9 GAL vps9:: kanMX* | Ungermann laboratory |
| CUY11101 | MATa *his3∆1 leu2∆0 met15∆0 ura3∆0 tor1::hphNT1* | Ungermann laboratory |
| CUY7894 | MATα *leu2-3, 112 ura3-52 his3-∆200 trp-∆901 lys2-801 suc2-∆9 GAL vps35::natNT2* | Ungermann laboratory |
| CUY8261 | MATα *leu2-3, 112 ura3-52 his3-∆200 trp-∆901 lys2-801 suc2-∆9 GAL vps5::natNT2* | Ungermann laboratory |
| CUY3252 | MATα *leu2-3, 112 ura3-52 his3-∆200 trp-∆901 lys2-801 suc2-∆9 GAL apl5::LEU2* | Ungermann laboratory |
| CUY12077 | MATa *his3∆1 leu2∆0 met15∆0 ura3∆0 fab1^S202A,S203A,S204A,T206A,S208A,S210A^* | Chen et al., 2021 |
| CUY4871 | MATa *his3∆200 leu2∆0 met15∆0 trp1∆63 ura3∆0 GYP7::HIS3MX6-GAL1pr* | Ungermann laboratory |
| CUY13395 | MATα *leu2-3,112 ura3-52 his3-∆200 trp-∆901 lys2-801 suc2-∆9 GAL VPS8::Chromobody-natNT2* | This study |
| CUY13338 | MATα *leu2-3,112 ura3-52 his3-∆200 trp-∆901 lys2-801 suc2-∆9 GAL GYP7::mGFP-hphNT1 VPS8::Chromobody-natNT2* | This study |
| CUY13948 | MATα *leu2-3,112 ura3-52 his3-∆200 trp-∆901 lys2-801 suc2-∆9 GAL ZRC1::Chromobody-natNT2* | This study |
| CUY13949 | MATα *leu2-3,112 ura3-52 his3-∆200 trp-∆901 lys2-801 suc2-∆9 GAL GYP7::mGFP-hphNT1 ZRC1::Chromobody-natNT2* | This study |
| CUY14214 | MATα *leu2-3,112 ura3-52 his3-∆200 trp-∆901 lys2-801 suc2-∆9 GAL GYP7::mGFP-hphNT1 ZRC1::Chromobody-natNT2 URA::pRS406-NOP1pr-YPT7 K127E* | This study |
| CUY14824 | MATα *leu2-3,112 ura3-52 his3-∆200 trp-∆901 lys2-801 suc2-∆9 GAL GYP7::mGFP-hphNT1 GYP7::GYP7(R458K)* | This study |
| CUY15030 | MATα *leu2-3,112 ura3-52 his3-∆200 trp-∆901 lys2-801 suc2-∆9 GAL GYP7::mGFP-hphNT1 GYP7::GYP7(R458K) ZRC1::Chromobody-natNT2* | This study |
| CUY15031 | MATα *leu2-3,112 ura3-52 his3-∆200 trp-∆901 lys2-801 suc2-∆9 GAL GYP7::mGFP-hphNT1 GYP7::GYP7(R458K) VPS8::Chromobody-natNT2* | This study |
| CUY11940 | MATα *leu2-3,112 ura3-52 his3-∆200 trp-∆901 lys2-801 suc2-∆9 GAL ypt7::natNT2 URA3::pRS406-YPT7pr-mNeon-(GGSG)x3-YPT7-YPT7term* | Langemeyer et al., 2020 |
| CUY12886 | MATα *leu2-3,112 ura3-52 his3-∆200 trp-∆901 lys2-801 suc2-∆9 GAL ypt7::natNT2 URA3::pRS406-YPT7pr-mNeon-(GGSG)x3-YPT7-YPT7term gyp7::hphNT1* | This study |
| CUY13952 | MATα *leu2-3,112 ura3-52 his3-∆200 trp-∆901 lys2-801 suc2-∆9 GAL ypt7::natNT2 URA3::pRS406-YPT7pr-mNeon-(GGSG)x3-YPT7-YPT7term GYP7::hphNT1-TEF1pr-3HA* | This study |
| CUY13443 | MATα *leu2-3,112 ura3-52 his3-∆200 trp-∆901 lys2-801 suc2-∆9 GAL ypt7::natNT2 URA3::pRS406-YPT7pr-mNeon-(GGSG)x3-YPT7-YPT7term MON1∆1-100::kanMX-MON1pr* | Borchers et al., 2023 |
| CUY13895 | MATα *leu2-3,112 ura3-52 his3-∆200 trp-∆901 lys2-801 suc2-∆9 GAL ypt7::natNT2 URA3::pRS406-YPT7pr-mNeon-(GGSG)x3-YPT7-YPT7term MON1∆1-100::kanMX-MON1pr gyp7::hphNT1* | This study |
| CUY13953 | MATα *leu2-3,112 ura3-52 his3-∆200 trp-∆901 lys2-801 suc2-∆9 GAL ypt7::natNT2 URA3::pRS406-YPT7pr-mNeon-(GGSG)x3-YPT7-YPT7term MON1∆1-100::kanMX-MON1pr GYP7::TEF1pr-3HA* | This study |
| CUY14682 | \|  \| MATα *leu2-3,112 ura3-52 his3-∆200 trp-∆901 lys2-801 suc2-∆9 GAL CCZ1::mNeon-natNT2 GYP7::2xmKate-HIS* \| \| --- \| --- \| | This study |
| CUY14683 | MATα *leu2-3,112 ura3-52 his3-∆200 trp-∆901 lys2-801 suc2-∆9 GAL MON1∆1-100::kanMX-Mon1pr CCZ1::mNeon-natNT2 GYP7::2xmKate-HIS* | This study |
| CUY14684 | \|  \| MATα *leu2-3,112 ura3-52 his3-∆200 trp-∆901 lys2-801 suc2-∆9 GAL GYP7::URA-TEFpr CCZ1::mNeon-natNT2 GYP7::2xmKate-HIS* \| \| --- \| --- \| | This study |
| CUY12969 | \|  \| MATα *leu2-3,112 ura3-52 his3-∆200 trp-∆901 lys2-801 suc2-∆9 GAL ypt7::natNT2 URA3::pRS406-YPT7pr-mNeon-(GGSG)x3-YPT7-YPT7term GYP7::2xmKate-hphNT1* \| \| --- \| --- \| | This study |
| CUY15036 | MATα *leu2-3,112 ura3-52 his3-∆200 trp-∆901 lys2-801 suc2-∆9 GAL ypt7::natNT2 URA3::pRS406-YPT7pr-mNeon-(GGSG)x3-YPT7-YPT7term MON1∆1-100::kanMX-Mon1pr GYP7::2xmKate-HIS* | This study |
| CUY15037 | \|  \| MATα *leu2-3,112 ura3-52 his3-∆200 trp-∆901 lys2-801 suc2-∆9 GAL ypt7∆::NATNT2 URA3::pRS406-YPT7pr-mNeon-(GGSG)x3-YPT7-YPT7term GYP7::hphNT1-TEFpr-3HA GYP7::2xmKate-HIS* \| \| --- \| --- \| | This study |
| CUY14676 | \|  \| MATα *leu2-3,112 ura3-52 his3-∆200 trp-∆901 lys2-801 suc2-∆9 GAL ypt7::natNT2 URA3::pRS406-YPT7pr-mNeon-(GGSG)x3-YPT7-YPT7term gyp7::hphNT1 msb3::kanMX* \| \| --- \| --- \| | This study |
| CUY14114 | MATα *leu2-3,112 ura3-52 his3-∆200 trp-∆901 lys2-801 suc2-∆9 GAL GYP7::URA-TEF1pr MUP1::GFP-natNT2* | This study |
| CUY13499 | MATα *leu2-3,112 ura3-52 his3-∆200 trp-∆901 lys2-801 suc2-∆9 GAL MON1∆1-100::kanMX-Mon1pr MUP1::GFP-hphNT1* | Borchers et al., 2023 or This study |
| CUY14112 | MATα *leu2-3,112 ura3-52 his3-∆200 trp-∆901 lys2-801 suc2-∆9 GAL MON1∆1-100::kanMX-MON1pr gyp7::hphNT1 MUP1::GFP-natNT2* | This study |
| CUY14113 | MATα *leu2-3,112 ura3-52 his3-∆200 trp-∆901 lys2-801 suc2-∆9 GAL MON1∆1-100::kanMX-MON1pr GYP7::TEFpr-3HA MUP1::GFP-natNT2* | This study |
| CUY14388 | MATα *leu2-3, 112 ura3-52 his3-∆200 trp-∆901 lys2-801 suc2-∆9 GAL ypt7∆::natNT2 URA3::pRS406-YPT7pr-mNeon-(GGSG)x3-YPT7-YPT7term VPS4::3xHA-mCherry-TRP* | This study |
| CUY14389 | MATα *leu2-3, 112 ura3-52 his3-∆200 trp-∆901 lys2-801 suc2-∆9 GAL ypt7::natNT2 URA3::pRS406-YPT7pr-mNeon-(GGSG)x3-YPT7-YPT7term GYP7::hphNT1-TEFpr-3HA VPS4::3xHA-mCherry-TRP* | This study |
| CUY14390 | MATα *leu2-3, 112 ura3-52 his3-∆200 trp-∆901 lys2-801 suc2-∆9 GAL ypt7::natNT2 URA3::pRS406-YPT7pr-mNeon-(GGSG)x3-YPT7-YPT7term VPS35::mKate-kanMX* | This study |
| CUY14391 | MATα *leu2-3, 112 ura3-52 his3-∆200 trp-∆901 lys2-801 suc2-∆9 GAL ypt7::natNT2 URA3::pRS406-YPT7pr-mNeon-(GGSG)x3-YPT7-YPT7term GYP7::hphNT1-TEFpr-3HA VPS35::mKate-kanMX* | This study |
| CUY14392 | MATα *leu2-3, 112 ura3-52 his3-∆200 trp-∆901 lys2-801 suc2-∆9 GAL ypt7::natNT2* *URA3::pRS406-YPT7pr-mNeon-(GGSG)x3-YPT7-YPT7term IVY1::mKate-kanMX* | This study |
| CUY14393 | MATα *leu2-3, 112 ura3-52 his3-∆200 trp-∆901 lys2-801 suc2-∆9 GAL ypt7::natNT2 URA3::pRS406-YPT7pr-mNeon-(GGSG)x3-YPT7-YPT7term GYP7::hphNT1-TEFpr-3HA IVY1::mKate-kanMX* | This study |
| CUY14394 | MATα *leu2-3, 112 ura3-52 his3-∆200 trp-∆901 lys2-801 suc2-∆9 GAL ypt7::natNT2 URA3::pRS406-YPT7pr-mNeon-(GGSG)x3-YPT7-YPT7term VPS21::kanMX-Pho5pr-mCherry* | This study |
| CUY14395 | MATα *leu2-3, 112 ura3-52 his3-∆200 trp-∆901 lys2-801 suc2-∆9 GAL ypt7::natNT2 URA3::pRS406-YPT7pr-mNeon-(GGSG)x3-YPT7-YPT7term GYP7::hphNT1-TEFpr-3HA VPS21::kanMX-Pho5pr-mCherry* | This study |
| CUY14495 | MATα *leu2-3,112 ura3-52 his3-∆200 trp-∆901 lys2-801 suc2-∆9 GAL TCO89::mNeon-natNT2* | This study |
| CUY14496 | MATα *leu2-3,112 ura3-52 his3-∆200 trp-∆901 lys2-801 suc2-∆9 GAL MON1∆1-100::kanMX-Mon1pr GYP7::hphNT1-TEFpr-3HA TCO89::mNeon-natNT2* | This study |
| CUY14503 | MATα *leu2-3,112 ura3-52 his3-∆200 trp-∆901 lys2-801 suc2-∆9 GAL PEP12::natNT2-Pho5pr-GFP* | This study |
| CUY14504 | MATα *leu2-3,112 ura3-52 his3-∆200 trp-∆901 lys2-801 suc2-∆9 GAL MON1∆1-100::kanMX-Mon1pr GYP7::hphNT1-TEFpr-3HA PEP12::natNT2-Pho5pr-GFP* | This study |
| CUY14590 | MATα *leu2-3,112 ura3-52 his3-∆200 trp-∆901 lys2-801 suc2-∆9 GAL GYP7::URA-TEFpr PEP12::natNT2-Phopr-GFP* | This study |
| CUY14694 | MATα *leu2-3,112 ura3-52 his3-∆200 trp-∆901 lys2-801 suc2-∆9 GAL MON1∆1-100::kanMX-Mon1pr Pep12::natNT2-Pho5pr-GFP* | This study |
| CUY14499 | MATα *leu2-3,112 ura3-52 his3-∆200 trp-∆901 lys2-801 suc2-∆9 GAL CPS1::URA-Pho5pr-GFP* | This study |
| CUY14500 | MATα *leu2-3,112 ura3-52 his3-∆200 trp-∆901 lys2-801 suc2-∆9 GAL MON1∆1-100::kanMX-Mon1pr GYP7::hphNT1-TEFpr-3HA CPS1::URA-Pho5pr-GFP* | This study |
| CUY3616 | MATα *leu2-3,112 ura3-52 his3-∆200 trp-∆901 lys2-801 suc2-∆9 GAL VPS21::kanMX-ADHpr VPS8::natNT2-TEFpr CPS1pr::URA3-PHO5pr-GFP* | Markgraf et al., 2009 |
| CUY14509 | MATα *leu2-3,112 ura3-52 his3-∆200 trp-∆901 lys2-801 suc2-∆9 GAL ypt7::natNT2 URA3::pRS406-YPT7pr-mNeon-(GGSG)x3-YPT7-YPT7term vps4::TRP* | This study |
| CUY14511 | MATα *leu2-3,112 ura3-52 his3-∆200 trp-∆901 lys2-801 suc2-∆9 GAL ypt7::natNT2 URA3::pRS406-YPT7pr-mNeon-(GGSG)x3-YPT7-YPT7term GYP7::hphNT1-TEFpr-3HA vps4::TRP* | This study |
| CUY4343 | MATα *his3∆200 leu2∆0 lys2∆0 met15∆0 trp1∆63 ura3∆0 YPT7::natNT2-TEF1pr-yeGFP* | Balderhaar et al., 2010 |
