# Supplementary material for: The GTPase activating protein Gyp7 regulates Rab7/Ypt7 activity on late endosomes
Source: J Cell Biol. 2024 Mar 27;223(6):e202305038. doi: 10.1083/jcb.202305038 (PMC10978497; doi:10.1083/jcb.202305038)
Supplement: Table S2 — lists plasmids used in this study. [file JCB_202305038_TableS2.docx]

**Table S2 Plasmids used in this study**

| **Protein** | **Backbone** | **Reference** |
| --- | --- | --- |
| Mrs6 | pET30 | Gift from K.Alexandrov |
| Bet4-Bet2 | pCDF-Duet-1 | Thomas et al., 2016 |
| GDI | pGEX-6P | Thomas et al., 2016 |
| Ypt7 | pET28d-GST-TEV | Lachmann et al., 2012 |
| Ypt7 | pET24b | Cabrera et al., 2014 |
| Gyp1-46 | pET22 | Wang et al., 2003 |
| Gyp7 | pQE32-His-TEV | Lachmann et al., 2012 |
| Gyp7 TBC | pET28a-His-SUMO | This study |
| Gyp7 PH | pET28a-His-SUMO | This study |
| Gyp7 PH+ | pET28a-His-SUMO | This study |
| Gyp7pr-Gyp7-mNeon | pRS405 | This study |
| NOP1pr-Ypt7 K127E | pRS406 | Cabrera and Ungermann, 2013 |
| Ypt7pr-mNeon-4x(GGSG)-Ypt7-Ypt7term | pRS406 | Langemeyer et al., 2020 |
| SCH9^709-824^-GFP-PHO8^1-63^ | pRS426-PRC1p-SCH9^709-824^-GFP-PHO8^1-63^ | Hatakeyama et al., 2019 |
| EEA1(human)^1257-1411^-GFP-SCH9^709-824^ | pRS425-VAC8p-EEA1(human)^1257-1411^-GFP-SCH9^709-824^ | Hatakeyama et al., 2019 |
| Gyp7 R458K | pRCC-K Gyp7 near 458aa | This study |
