# Supplementary material for: The GTPase activating protein Gyp7 regulates Rab7/Ypt7 activity on late endosomes
Source: J Cell Biol. 2024 Mar 27;223(6):e202305038. doi: 10.1083/jcb.202305038 (PMC10978497; doi:10.1083/jcb.202305038)
Supplement: Table S3 — lists primers used in this study. [file JCB_202305038_TableS3.docx]

**Table S3 Primers used in this study**

| **Name** | **5’-3’Sequence** |
| --- | --- |
| Gyp7 S2 | CGAATTATATCTCCGGTATTCAATATGTAAAGTTCCGTTTCTATTTACCACTAATCGATGAATTCGAGCTCG |
| Gyp7 S3 | CCTATCATAAGACATGAAGGGCAAAGGAGCAAAAATTCCGTTAAACGTACGCTGCAGGTCGAC |
| Vps35 S3 | GAAAGTCAAAGAGAAGTTGACGATCGTTTCAAAGTCATATATGTACGTACGCTGCAGGTCGAC |
| Vps35 S2 | GTAGTTTTTTTTTATCTTGGGCATGTACGAAGAGCAAGTACGTTATTTAACTAATCGATGAATTCGAGCTCG |
| Vps21 S4 | ACTGCTGCCTCACCCAACAGTACCAACTTTATGGAAGTGACTGATGTGTTCATCGATGAATTCTCTGTCG |
| Vps21 S1 | CGACTTATCTAAGCTATAAAAAAATATCCCTTTTATCACACAAAAAAATGCGTACGCTGCAGGTCGAC |
| Muk1 S1 | GCCAAGAAGAAGAGCAGATAAAAAAGAAGTACGCCCTCTAACAATAAAAGTCAAATGCGTACGCTGCAGGTCGAC |
| Muk1 S2 | CCTTCTCTCCTCCTCTTTCGCATCTATACATACGATTTCATATATACGTTTCAATCGATGAATTCGAGCTCG |
| Ypt52 S1 | GGAAGAAGGTTTAACTTATTTGGAGTAAACGTATATATTATTAACAGATGCGTACGCTGCAGGTCGAC |
| Ypt52 S2 | TAAAGCAACTTCTGTTGTTTTTCCTCTAAAACACAAATCATAGGATATCAATCGATGAATTCGAGCTCG |
| Msb3 S3 | GCATCAAGGAGAAAGTGAAGCAGATGAGGAAGAAAAAAGACAAGGGTGACCGTACGCTGCAGGTCGAC |
| Msb3 S2 | AATTATCACATATTTGTTTATGCAAAAACAAAAACAGGAAGCAAAAGTTAATCGATGAATTCGAGCTCG |
| Gyp7 S1 | GTTGCACAAATTGGCAAAGTTCTACAAGAGTCATTCATACATCCCCTGATGCGTACGCTGCAGGTCGAC |
| Gyp7 S4 | TCG CTT GTT GGG TGT AAG AAC ACT TTA GAT TTG CAG AAT AGT ATC TTA CTC ATC GAT GAA TTC TCT GTC G |
| Vps39 S1 | GTCTTATATTGATCAGCAAAAACCCTTCAAAATATCAATTTATACCAAAAATTAAATGCGTACGCTGCAGGTCGAC |
| Vps39 S4 | GCAAAATCGCAGTGATATCCGATGATTTCAGCGAGTGTAGCTTTTGAGCTCTTAACATCGATGAATTCTCTGTCG |
| 5’ Vps45 ko (pRS400) | ACAGTGACTTGGTTTTGAGTTAAGGCCATCTTTTACTGTATAAGATTGTACTGAGAGTGCAC |
| 3’ Vps45 ko (pRS400) | CCTCATATATAAAATAGAATTTTAGAATAAGATAATCCTTATCTGTGCGG  TATTTCACACCG |
| Vps3 S1 | CAGTCAAGGAGACTACCTTTTTTTGGTTGCAACCATAATATTATAGAACCATGCGTACGCTGCAGGTCGAC |
| Vps3 S2 | ATAAAATTTGAATTGTATGCCTGAACAGAAAAAGAATAGGTGGGCTCTTCATCGATGAATTCGAGCTCG |
| Vps35 S1 | ATAACGATAAAAGGAGGAGGACGAGAAAGAAGAAGCTGAAAAACACAATG CGTACGCTGCAGGTCGAC |
| Vps5 S2 | TAAATCCTGAGGAACGTGACACATAAAGTTATTGTATACAGATCATCTAAATCGATGAATTCGAGCTCG |
| Vps5 S1 | TGCAGCAGGGATTTTATAAACTTTCATACATCCTGCAATAACAAGCCATG CGTACGCTGCAGGTCGAC |
| Mvp1 S1 | CGAATTTGGATTCTATAAAACACCACTGAGGCGAAAAAAAAAGTAATGCGTACGCTGCAGGTCGAC |
| Mvp1 S2 | CAATAATGTTTTGGCAGACTAAGTGGTTAGTCTTCACTCCGTAAAATTTAATCGATGAATTCGAGCTCG |
| Snx4 S1 | AAACTTTATTTACGGTATACCACAATACTGCTCTTTTTGTTGAGGATATGCGTACGCTGCAGGTCGAC |
| Snx4 S2 | GTGCCCAAGGTATTATCAGTAGTAATGGGAAAACATTAAGAGCACCACTAATCGATGAATTCGAGCTCG |
| Vps38 S1 | GAATTGATGGTTTTACCTATTAGGGATAGTAATCATAATTTAAAAATATGCGTACGCTGCAGGTCGAC |
| Vps38 S2 | CATGGAAAAGATTAAATGGCAGTCCAAAAGAGATTTTTGATTTTCAGTCTAATCGATGAATTCGAGCTCG |
| Mup1 S2 | GTTCATACGTGATTATAAGAATCGAGATGAGATGGTAAGTACCTTTTTGGTTAATCGATGAATTCGAGCTCG |
| Mup1 S3 | CGTTATTGAAACGAATATAATCGAACATTACAAAAGTGAACAAGAAAAATCGCTGCGTACGCTGCAGGTCGAC |
| Vps8 S3 | ATGAATATTCTTGTTTAATTTGCCAGACGGAATCTAACCCAAAAATAGTACGTACGCTGCAGGTCGAC |
| Vps8 S2 | TATAAATTTTACTTTTATGTAACCAAAGTTGTATTAAATATTTAGAAATGATCGATGAATTCGAGCTCG |
| Zrc1 S2 | CTGTAGAACCATGGGATAAATTCACCGACGGCTTCAGCCCTTTATATCTTTATTAATCGATGAATTCGAGCTCG |
| Zrc1 S3 | GCATCATCTAATTGTATTGTAGATGACGCTGTAAACTGCAATACTTCCAATTGCCTGCGTACGCTGCAGGTCGAC |
| 5’ Gyp7 near 458aa Proto-F | GAGACGCTGTGATAGAAACTGTTTTAGAGCTAGAAATAGCAAGTTAAAATAAGG |
| 3’ Gyp7 near 458aa Proto-R | AGTTTCTATCACAGCGTCTCGATCATTTATCTTTCACTGCGGAG |
| Msb3 S1 | CTTTGTAGGAGTCAAAGAGTTGCGCACCCAGAACCATTGTAATTAGCATGCGTACGCTGCAGGTCGAC |
| 5´Vps4-3HA-eGFP-longtine | CTTGCTGAAGCAAGAACAGTTCACTAGAGATTTTGGTCAAGAAGGTGGATCCCCCGGGCGCATCTT |
| 3´Vps4-3HA-eGFP-longtine | GTTTCTAAGTAAAGGGAAGAGTTAACTGTCCTCTGCTTTTCTTTATCGAATTCGAGCTCGTTTAAAC |
| Ivy1 S3 | ACAATCACAGCAGCGATACGGACGGCATGCAAGACCAGTCAAGTAATATA  CGTACGCTGCAGGTCGAC |
| Ivy1 S2 | TGTTCACTTTCTCCATTTCTATATAAAAAGCATACATAGAGTTACAAATT  ATCGATGAATTCGAGCTCG |
| Tco89 S2 | CCTCGAAAGCATTAGCTACTCTTTTAAACTGTGTGCTTCGTGTTGGTTGTTTTCAATCGATGAATTCGAGCTCG |
| Tco89 S3 | ATCGCAGGATGGAAAATGTGGGCTACATGCATACACAGCCACAACAAAGGCGTACGCTGCAGGTCGAC |
| Pep12 S1 | CTTTTATAGAAGAATATAACGTAAATTACTACAATAATTGTGTTGAGATGCGTACGCTGCAGGTCGAC |
| Pep12 S4 | GAACCGTTCCAAACGGCTTCATTATCACCACCAAAAAATTCGTCTTCCGACATCGATGAATTCTCTGTCG |
| 5’ Cps1 Munro tagging | ACTAATCCTGCATCATCACATTAAGGAATCATTCATCTAACAATTACATTGATTGTACTGAGAGTGCACC |
| 3’ Cps1 Munro tagging | CTTTGCCATAGGGACTTTCTAGGGGCCTTCTCTACTGGTAAGGCGATCATAGATCTCAAGTCCTCTTCAGA |
| 5’ Vps4 ko (pRS400) | GGAAGACAAAAATAAAGCATAGAGTGCCTATAGTAGATGGGGTACAAAGATTGTACTGAGAGTGCAC |
| 3’ Vps4 ko (pRS400) | ATTTTTTATTTTCATGTACACAAGAAATCTACATTAGCACGTTAATCA ATCTGTGCGGTATTTCACACCG |
| 5‘-Gyp7 4xGSS aa321-706 for | GCGGATCCGGGTCCTCCGGCTCGTCCGGAAGTAGTGGGTCTTCTTTTGATTCGTATAGGAATAACATTTTC |
| 5‘-Gyp7 aa321-706 rev | GGCCTCGAGTTAGGATACGTTTTGTAAATCTCTTTCC |
| 5‘-Gyp7 4xGSS aa1-197 for | GCGGATCCGGGTCCTCCGGCTCGTCCGGAAGTAGTGGGTCTTCTATGAGTAAGATACTATTCTGC |
| 5‘-Gyp7 aa1-197 rev | GGCCTCGAGTTAGGGATTTACTAACCAAAACTC |
| 5’-Gyp7 aa1-205 rev | GGCCTCGAGTTAGAAATTTCTCAAATCATTCAAAG |
| 5’ BamHI-Gyp7 | ATGATGGATCCATGAGTAAGATACTATTCTGC |
| 3’ XbaI-Gyp7 (w/o STOP) | GGCTCTAGATTTAACGGAATTTTTGCTCCTTTGCCC |
