# Supplementary figures and images for: The GTPase activating protein Gyp7 regulates Rab7/Ypt7 activity on late endosomes
Source: J Cell Biol. 2024 Mar 27;223(6):e202305038. doi: 10.1083/jcb.202305038 (PMC10978497; doi:10.1083/jcb.202305038)

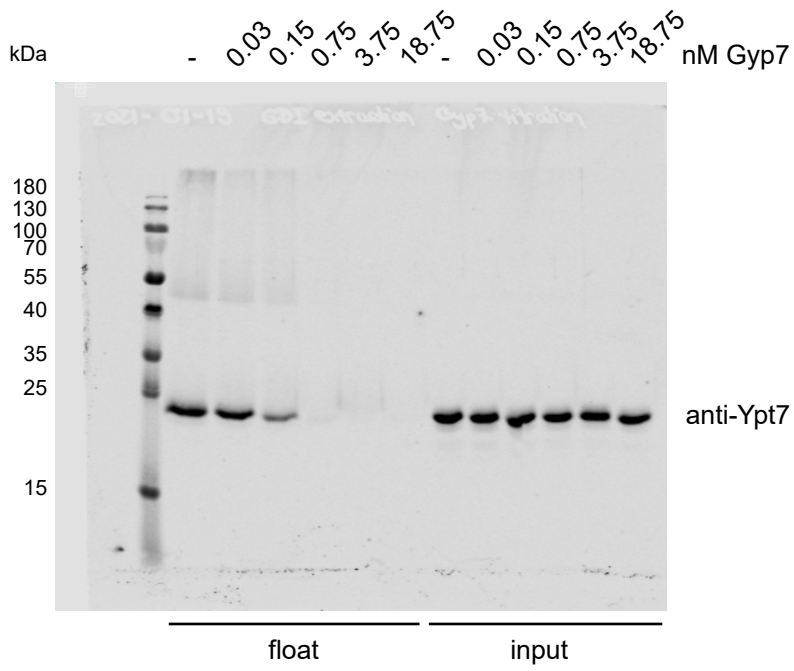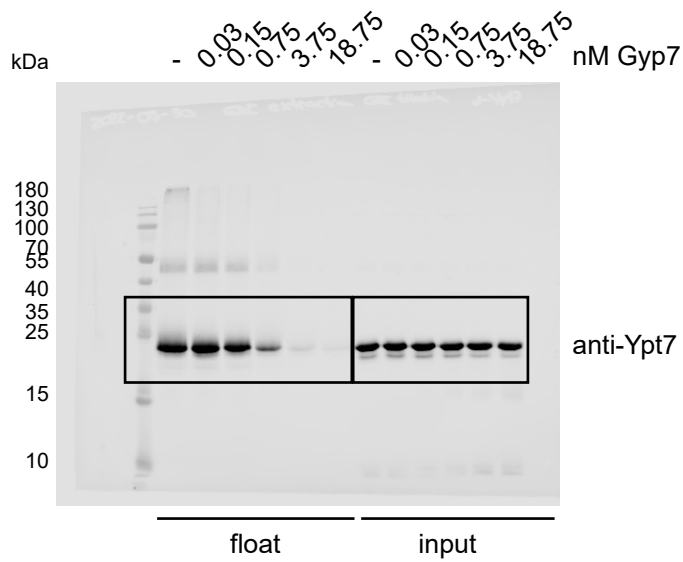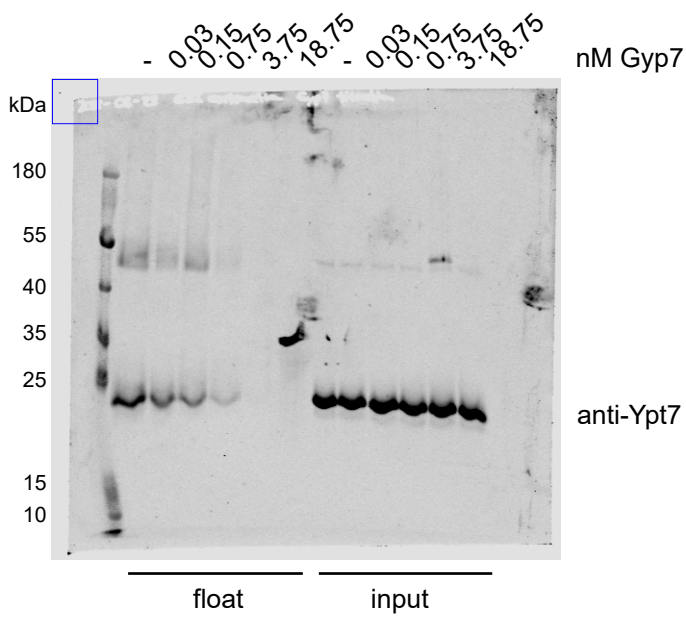

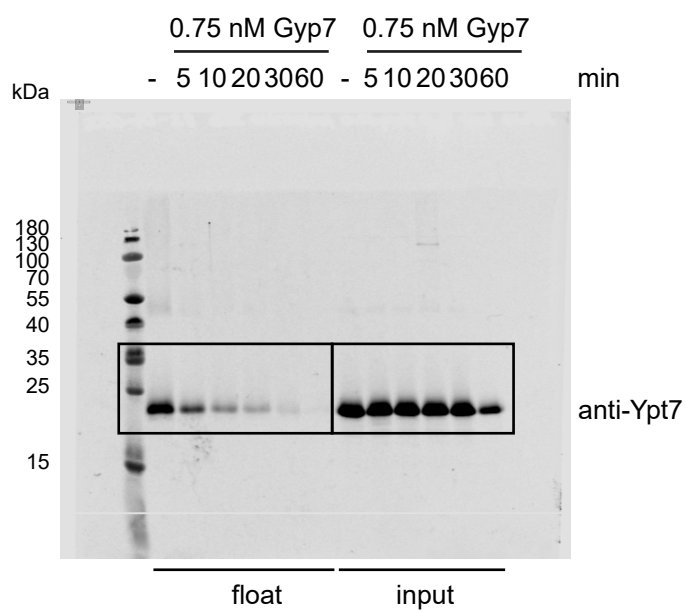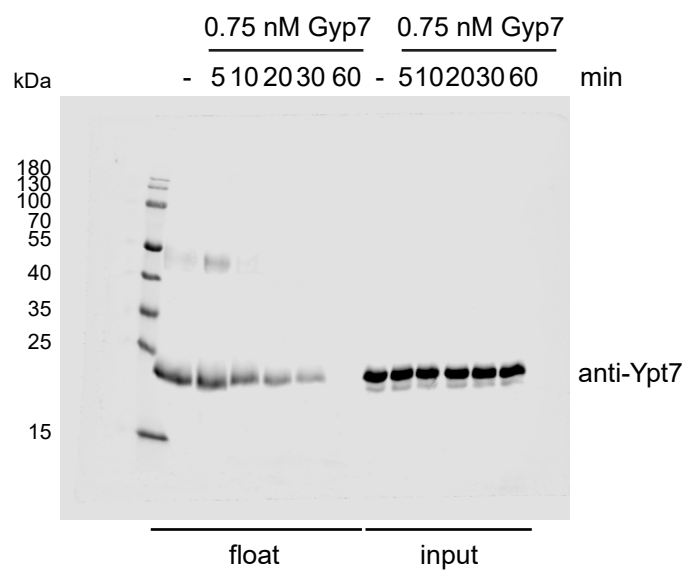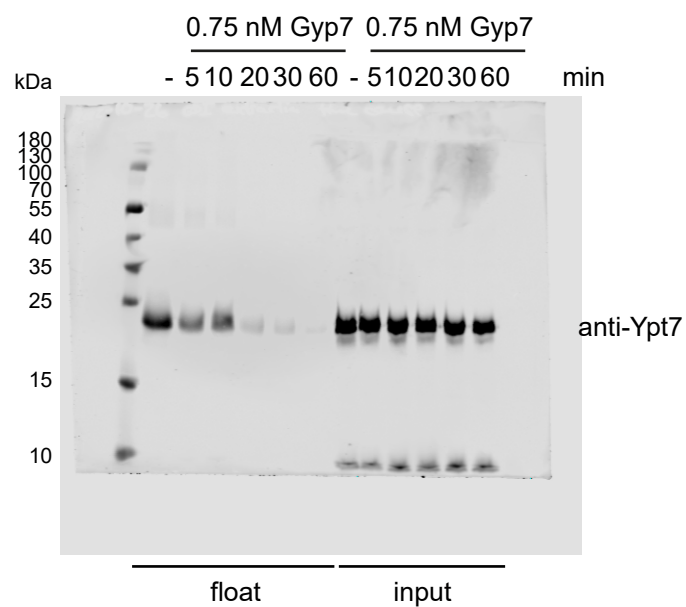

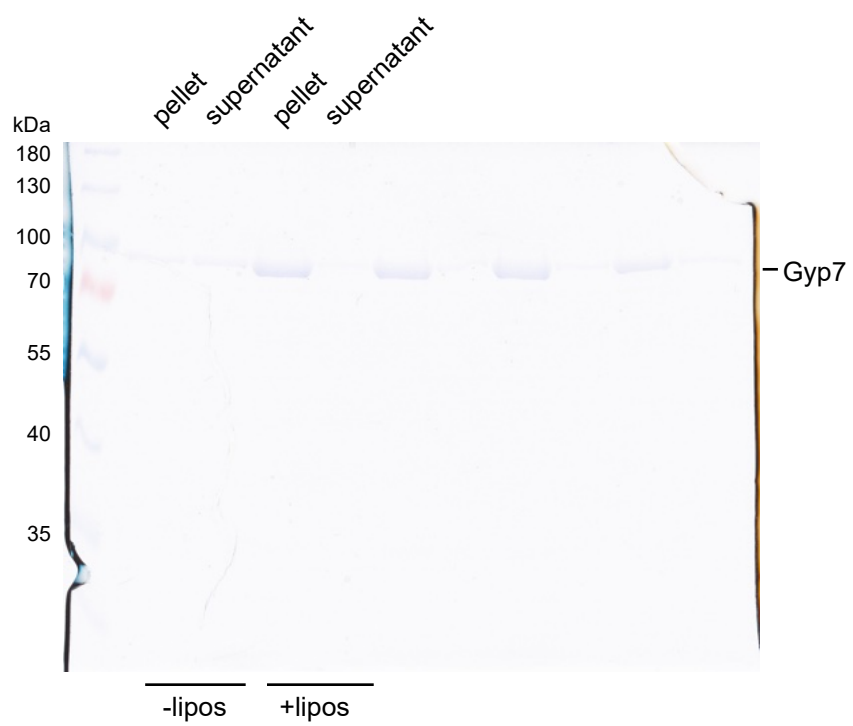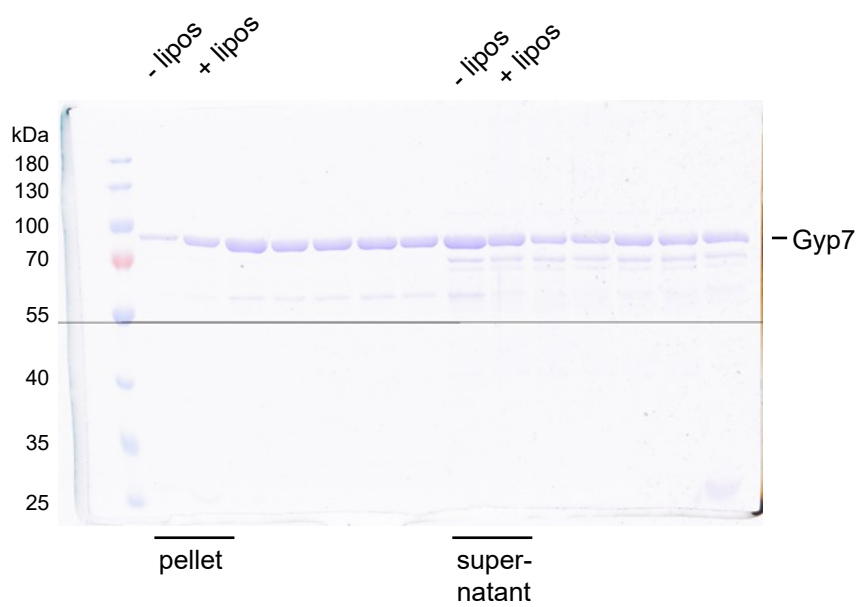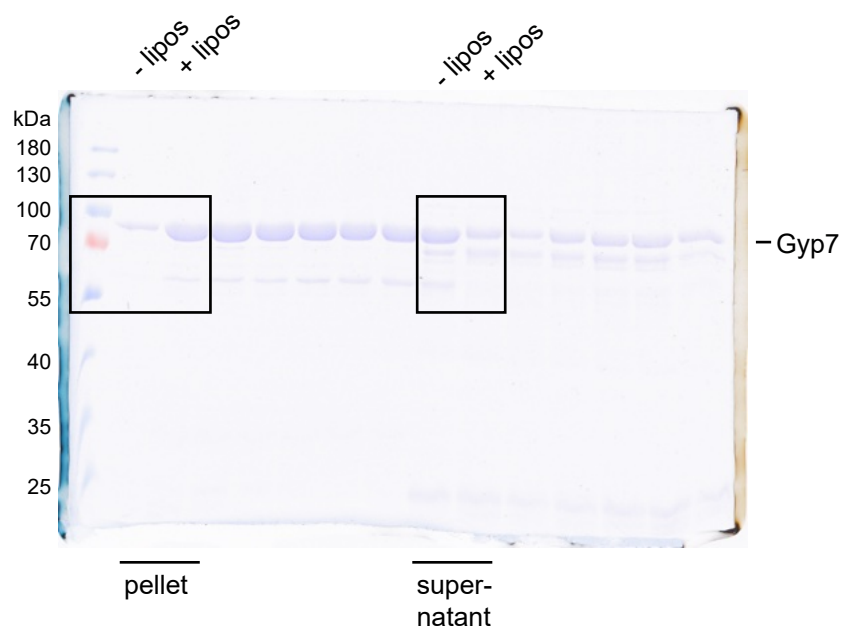

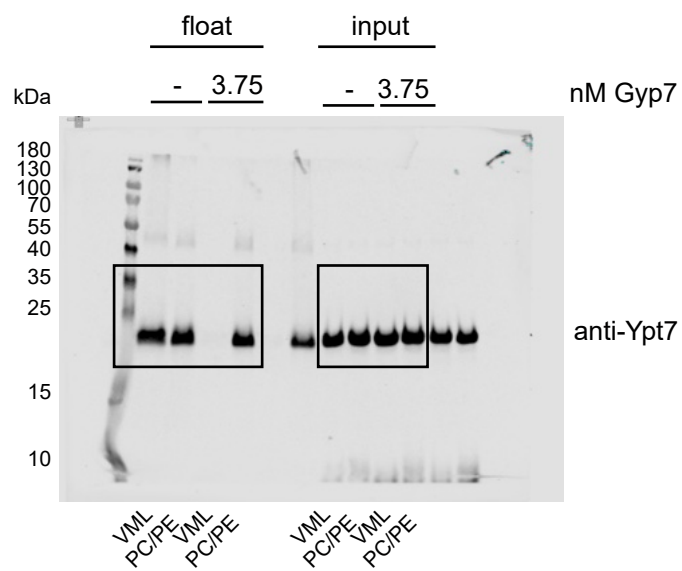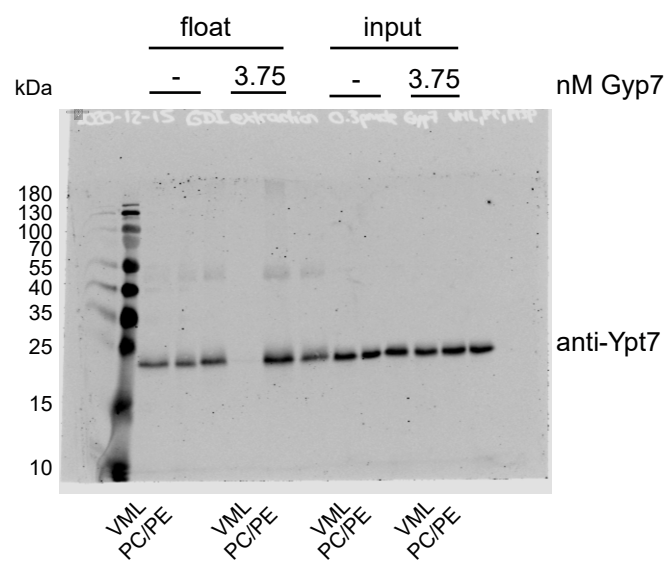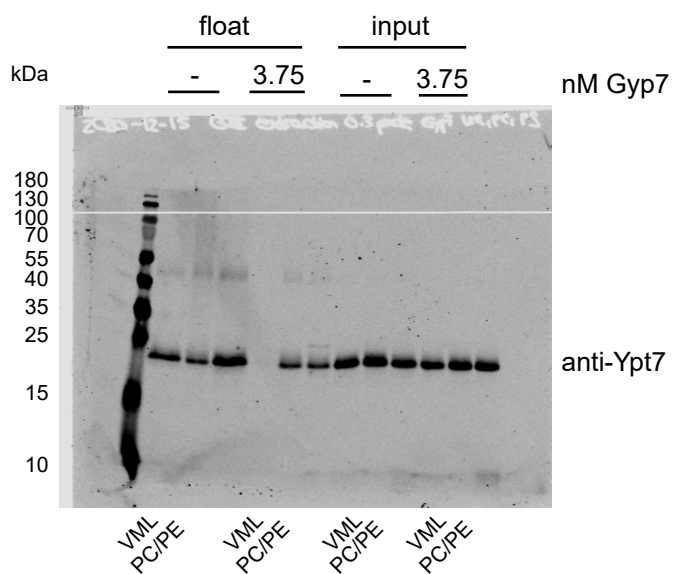

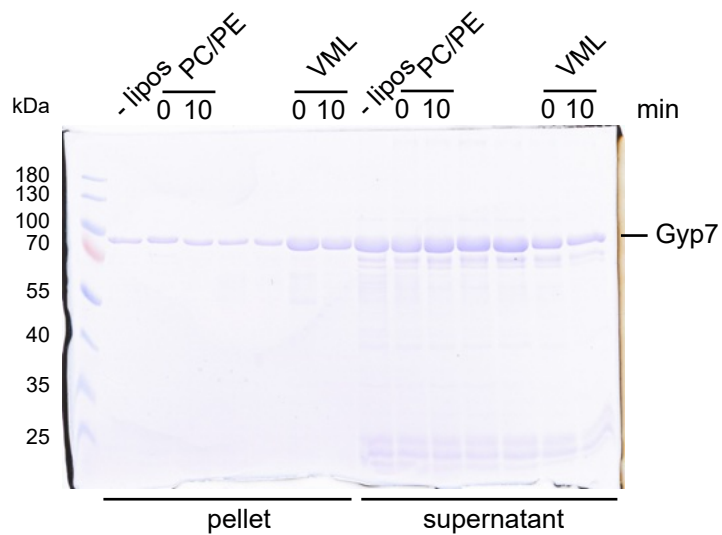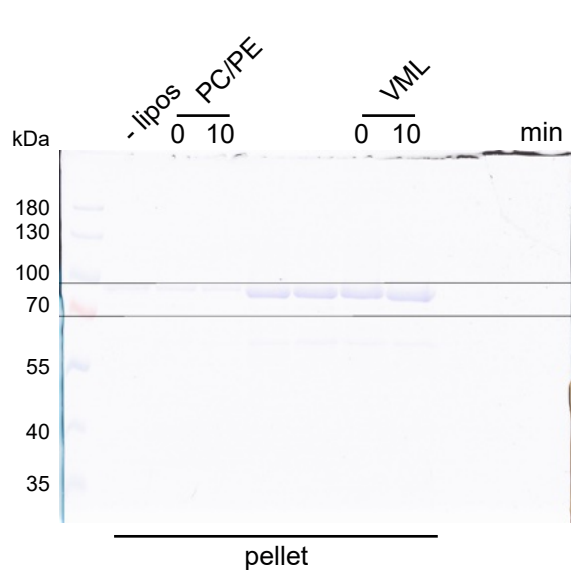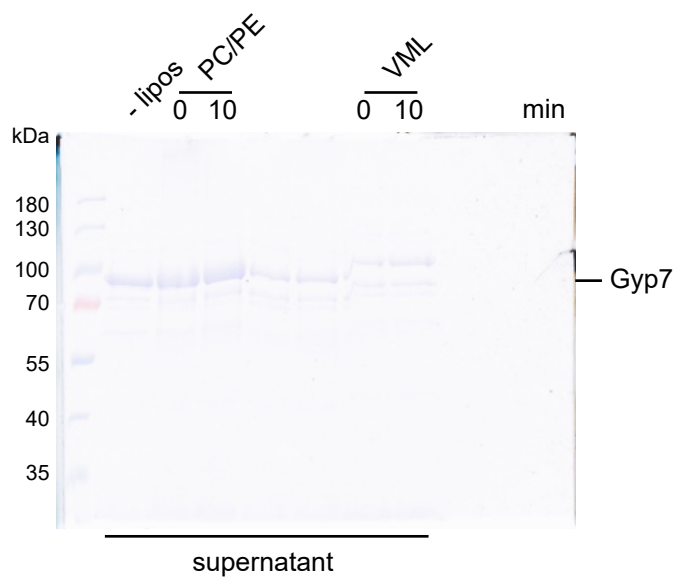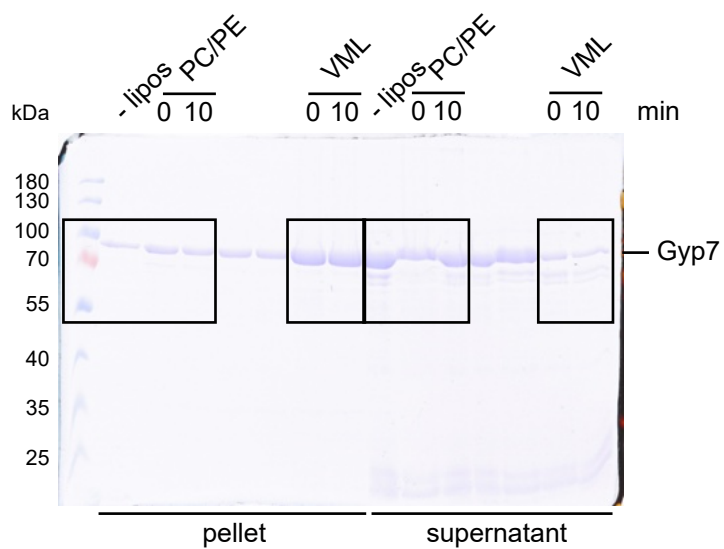

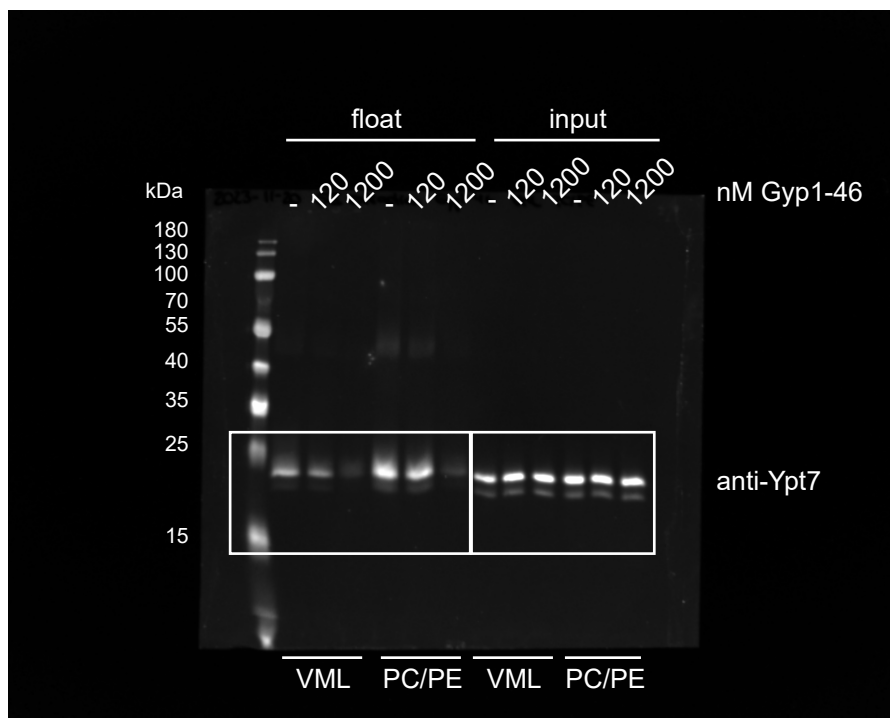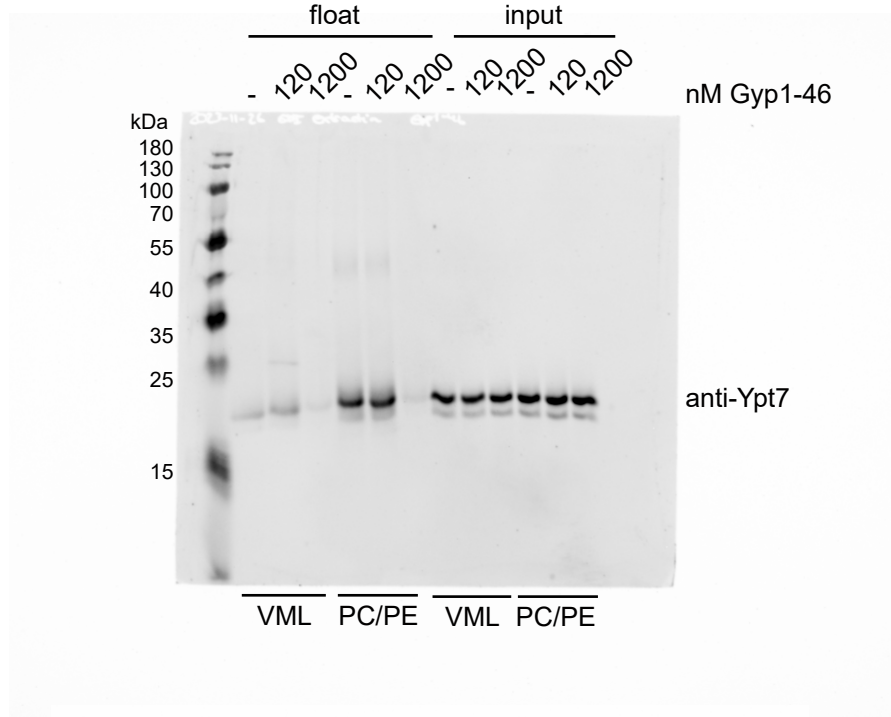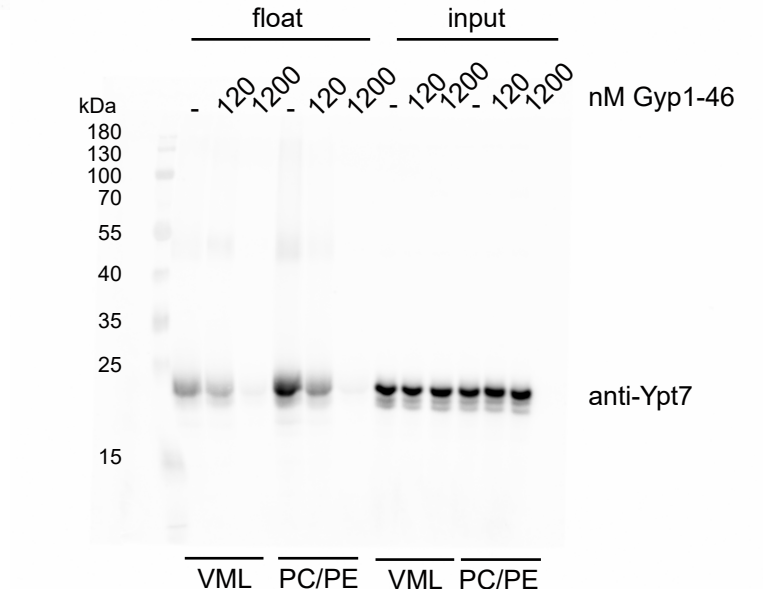

Supplement: SourceData F4 — is the source file for Fig. 4. [file JCB_202305038_SourceDataF4.pdf]

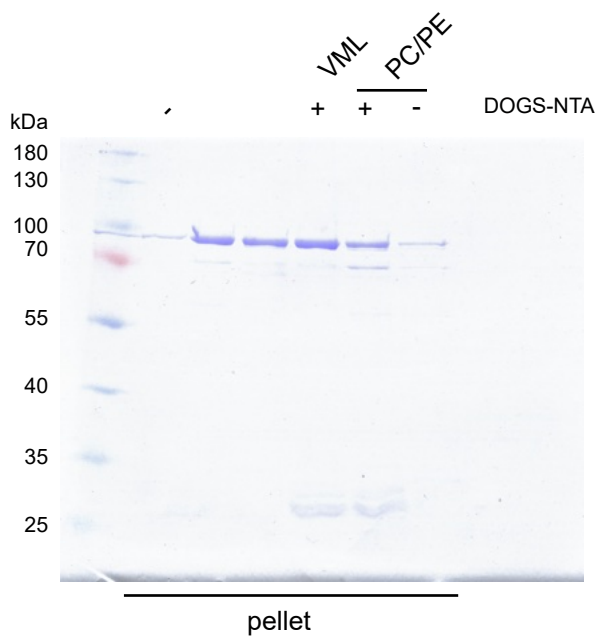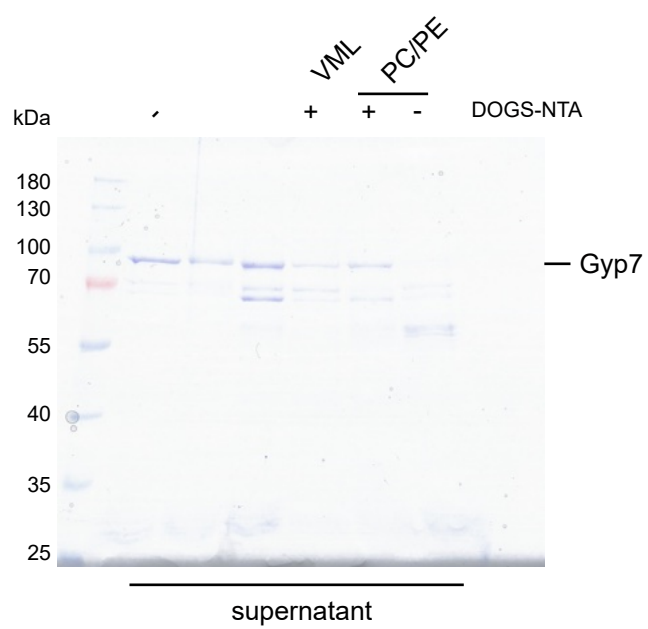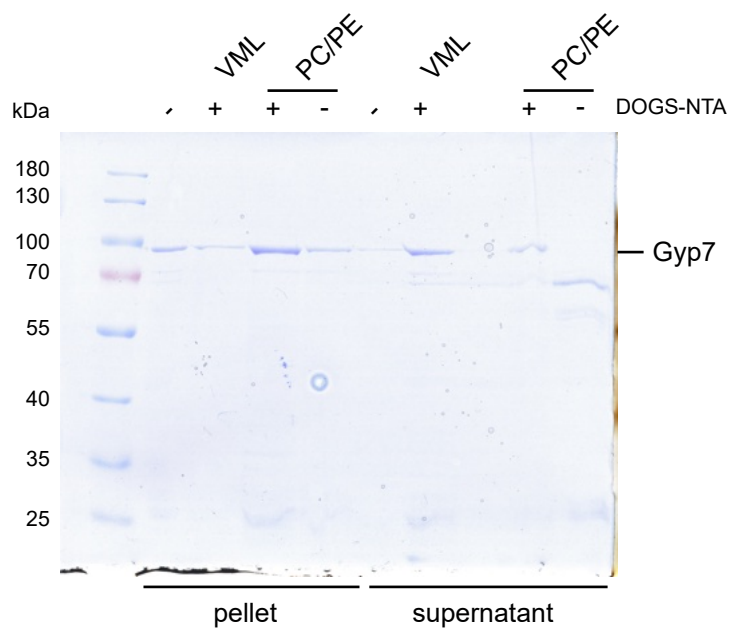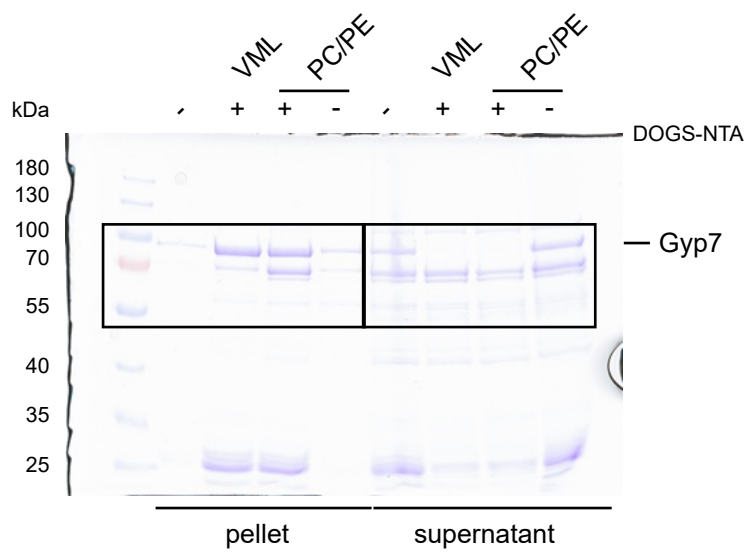

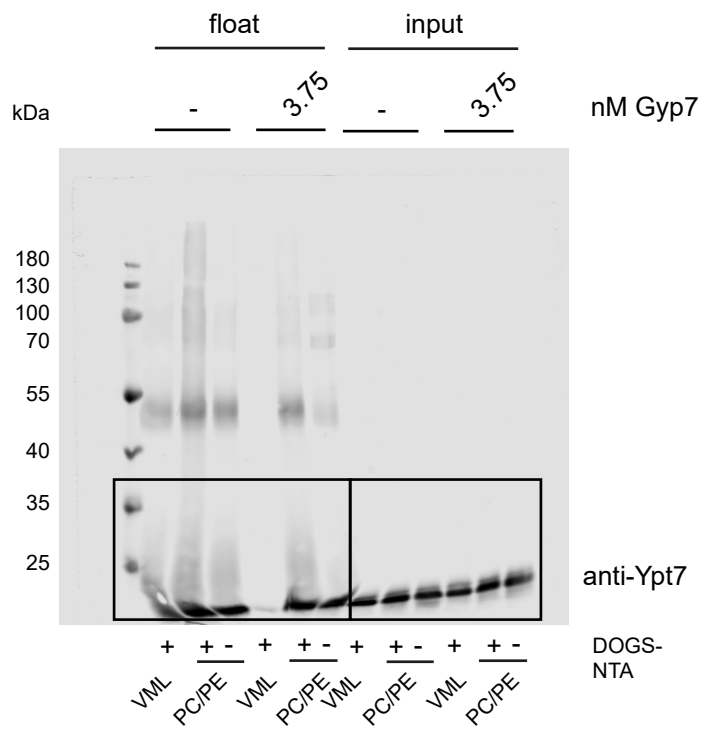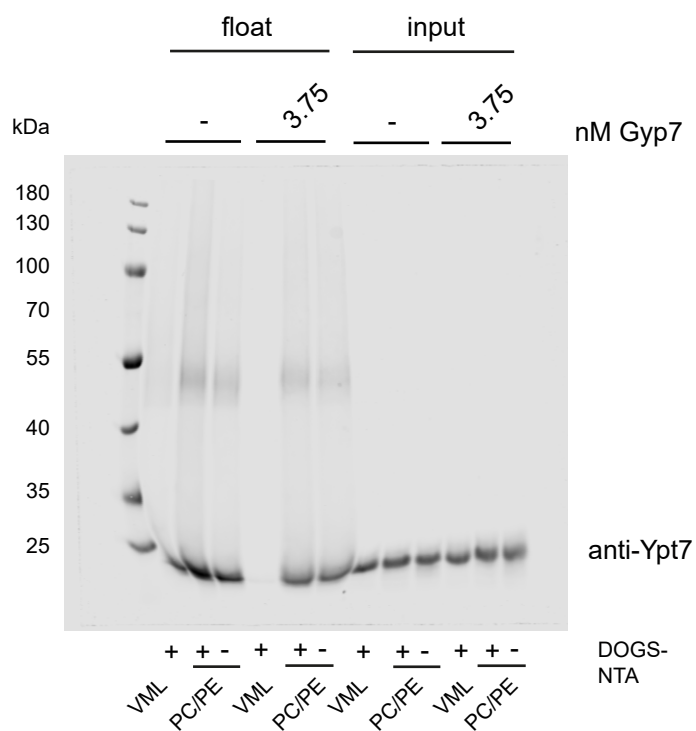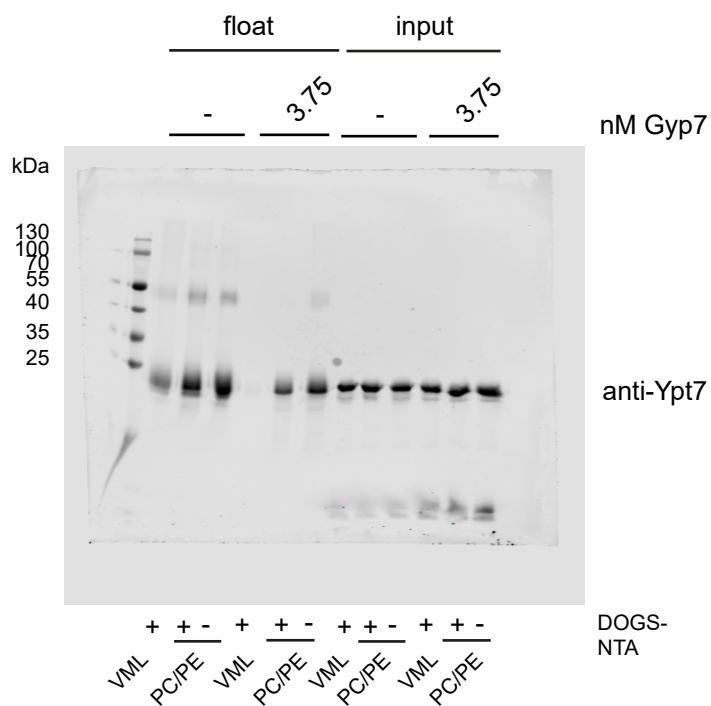

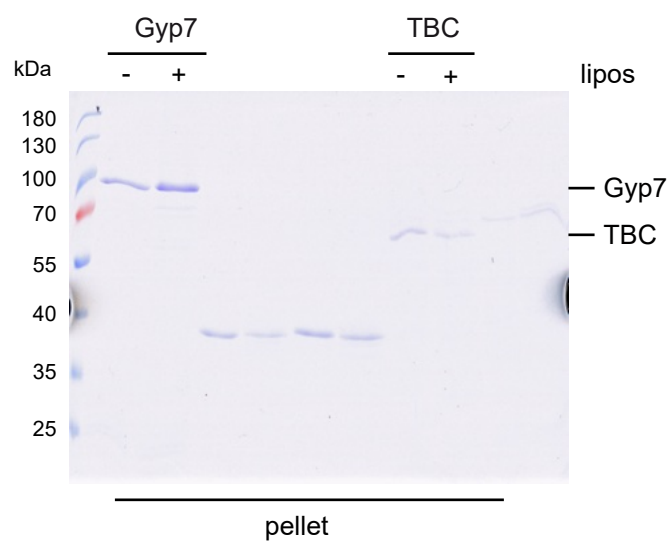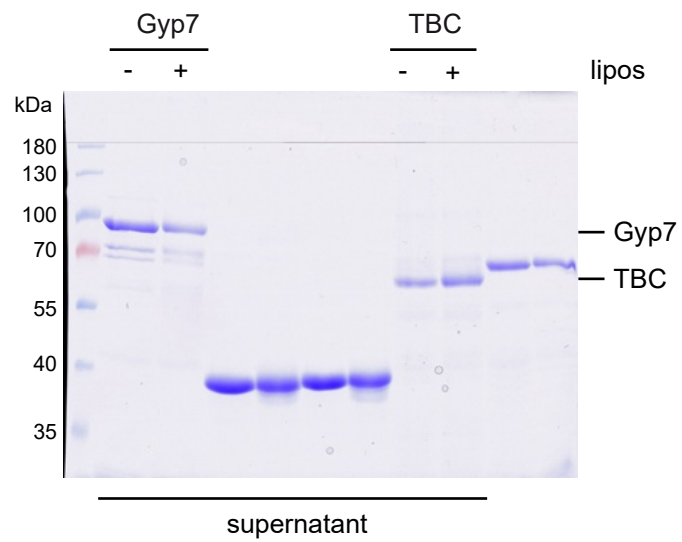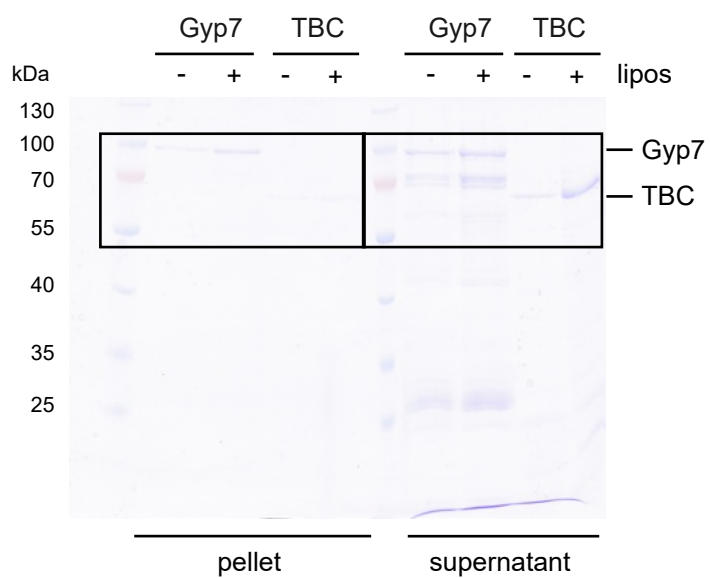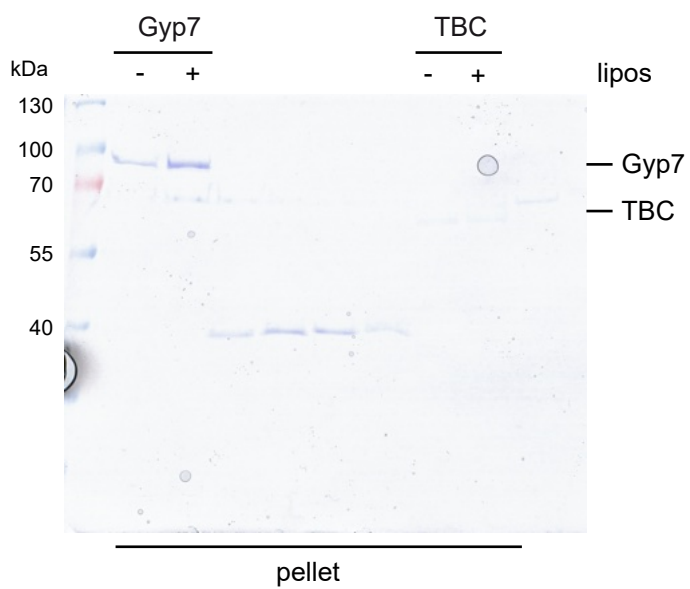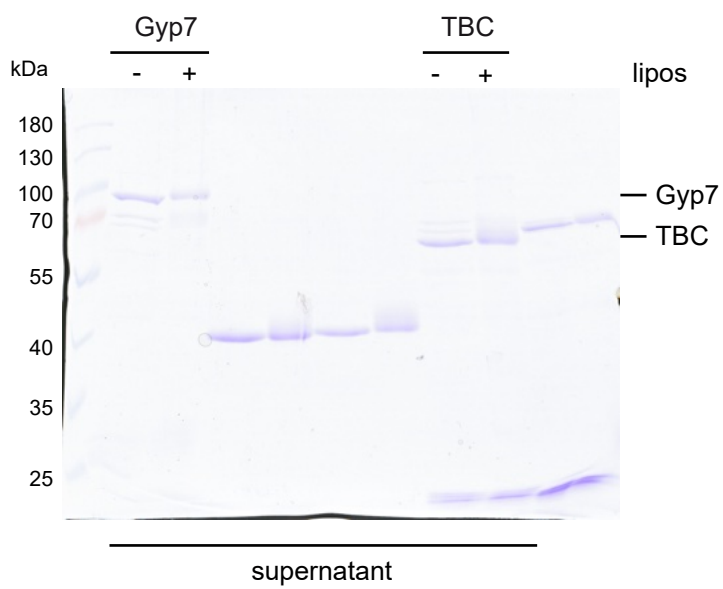

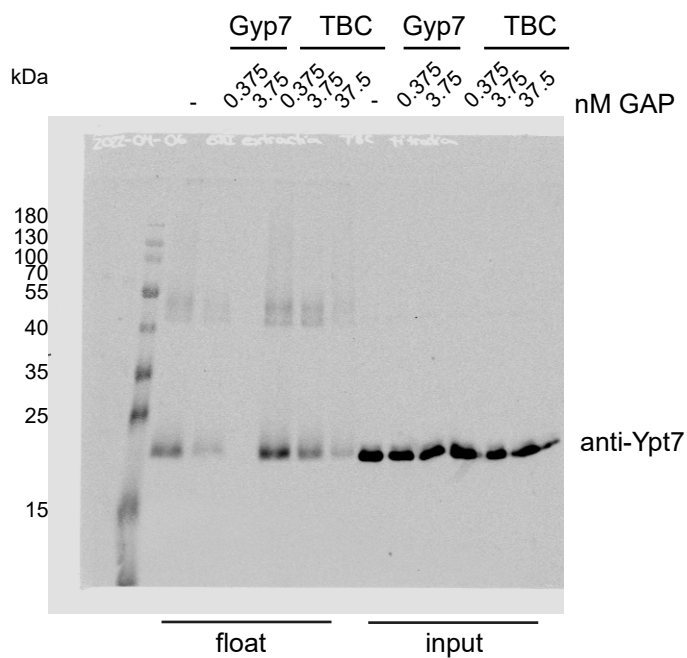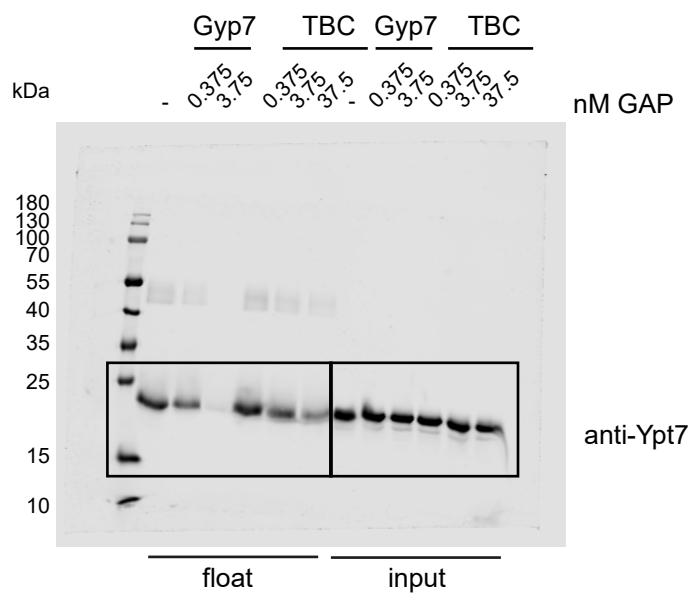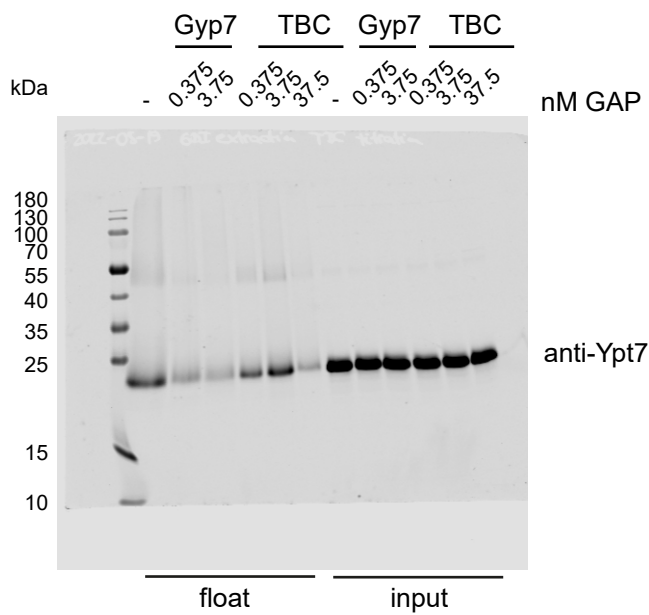

Supplement: SourceData F5 — is the source file for Fig. 5. [file JCB_202305038_SourceDataF5.pdf]

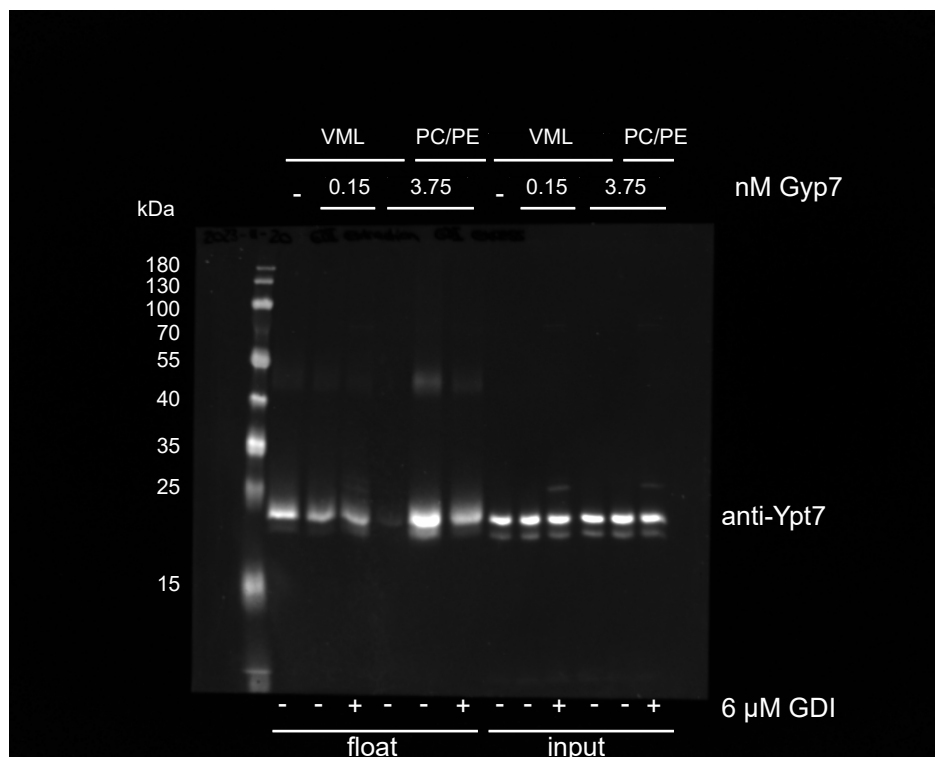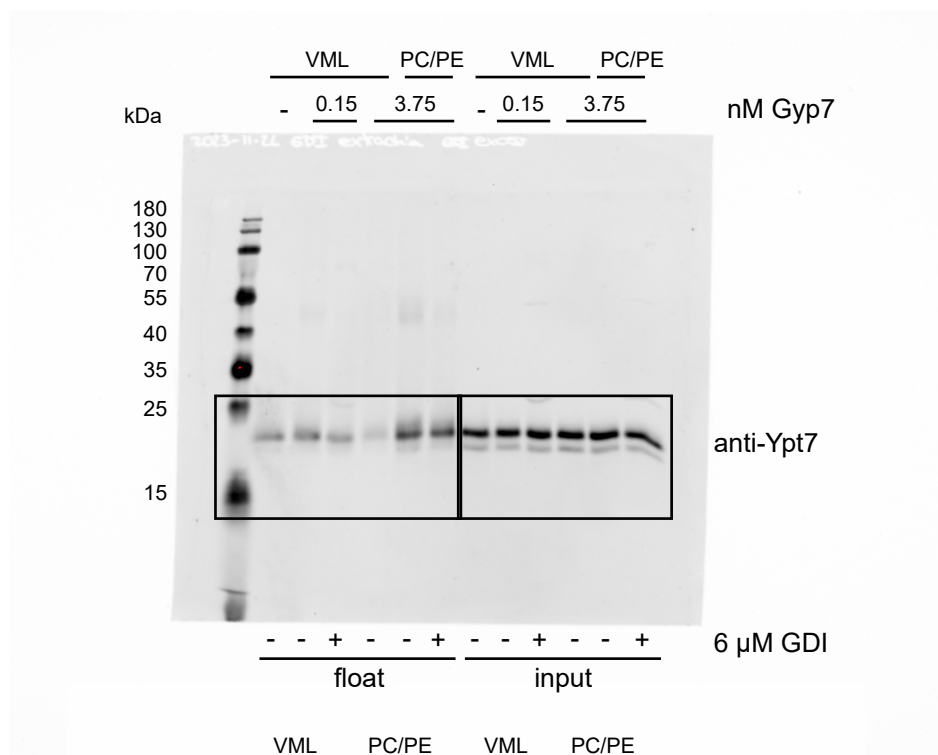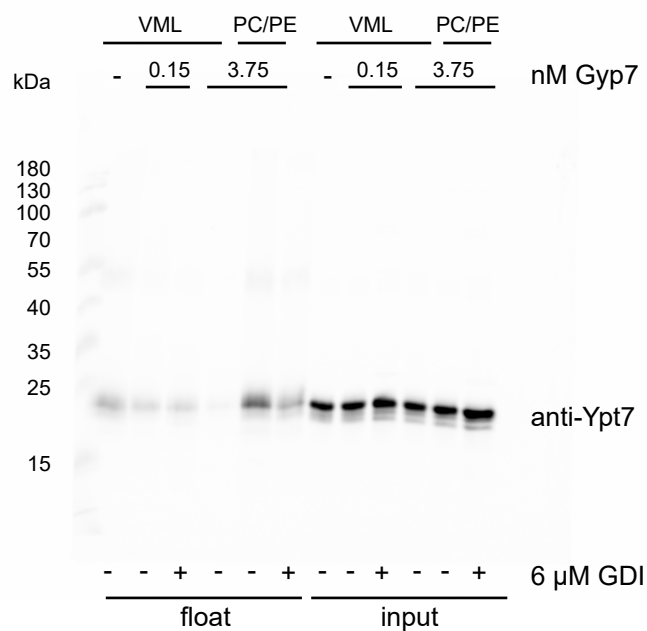

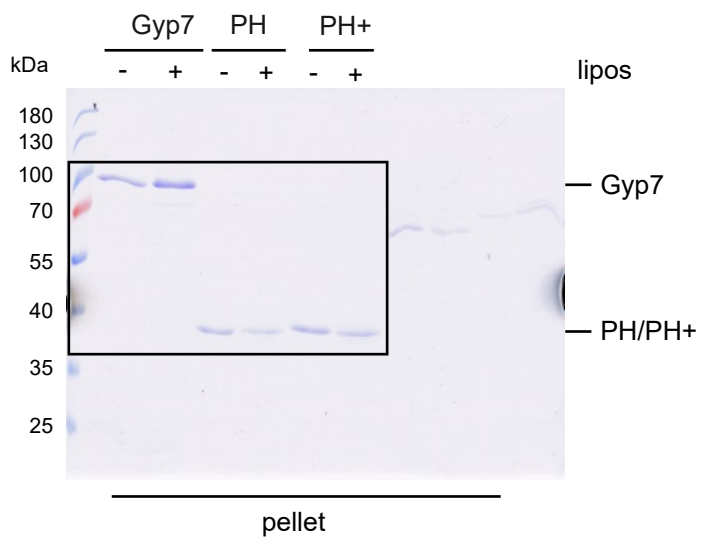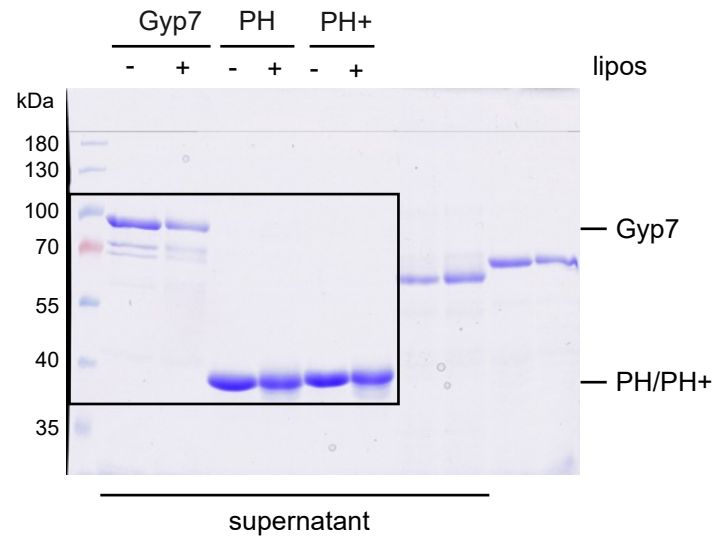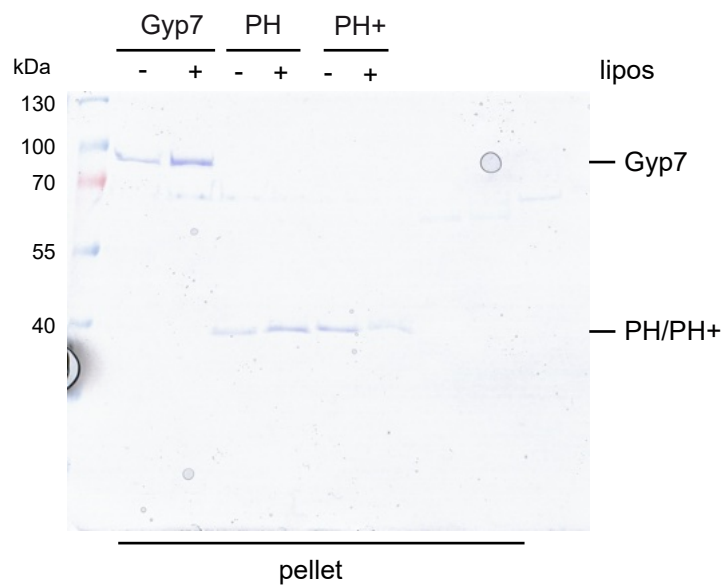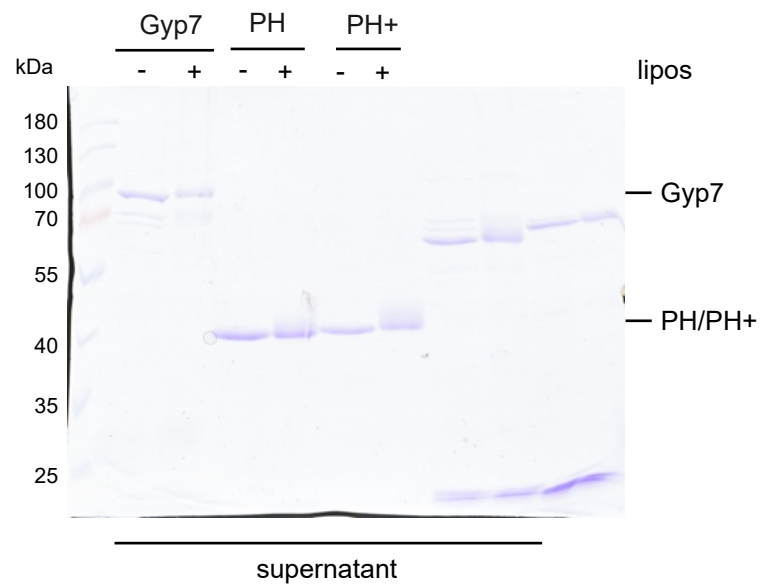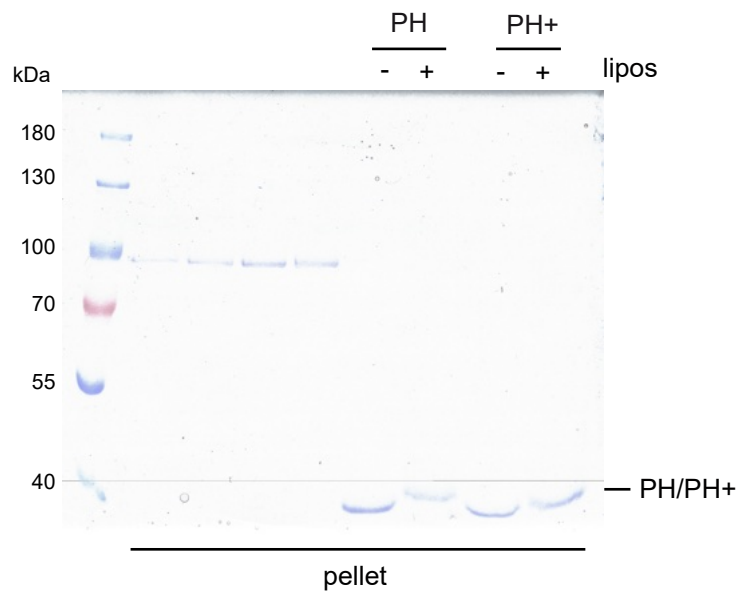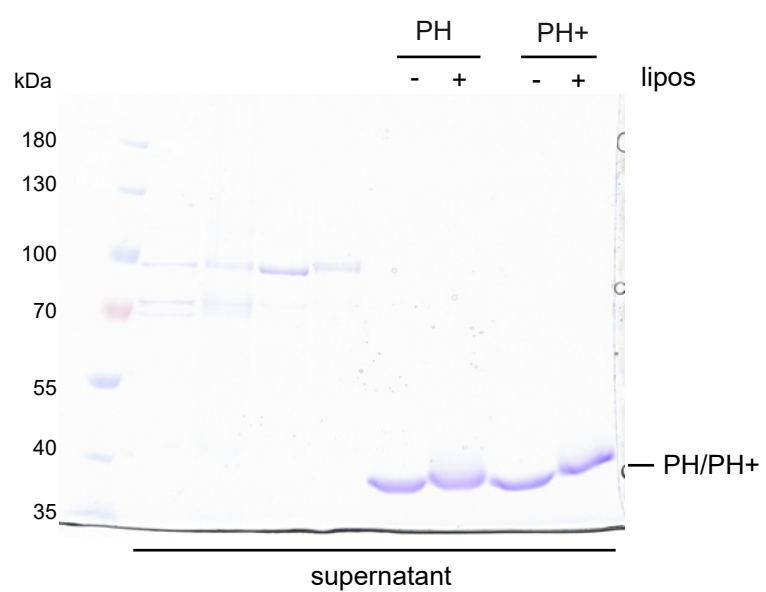

Supplement: SourceData FS3 — is the source file for Fig. S3. [file JCB_202305038_SourceDataFS3.pdf]

mNeon-Ypt7

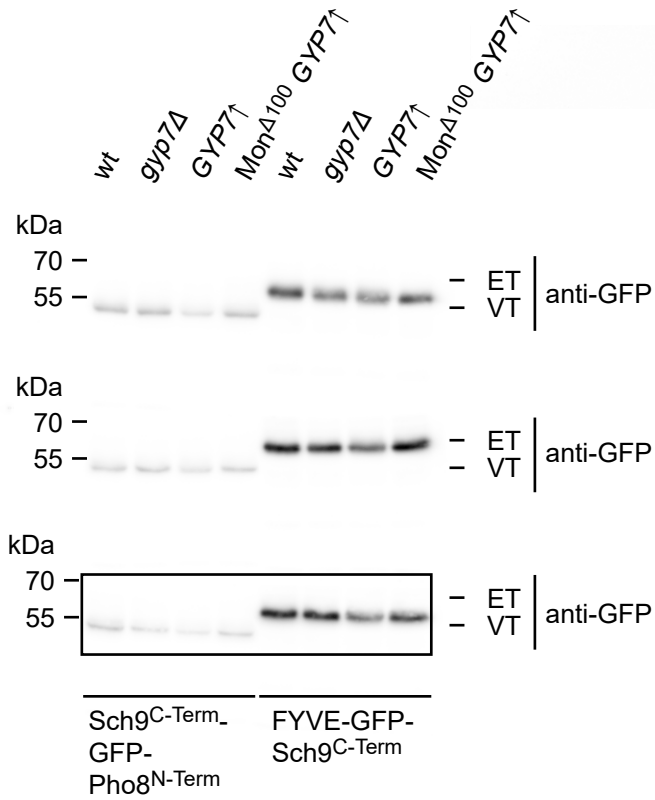

mNeon-Ypt7

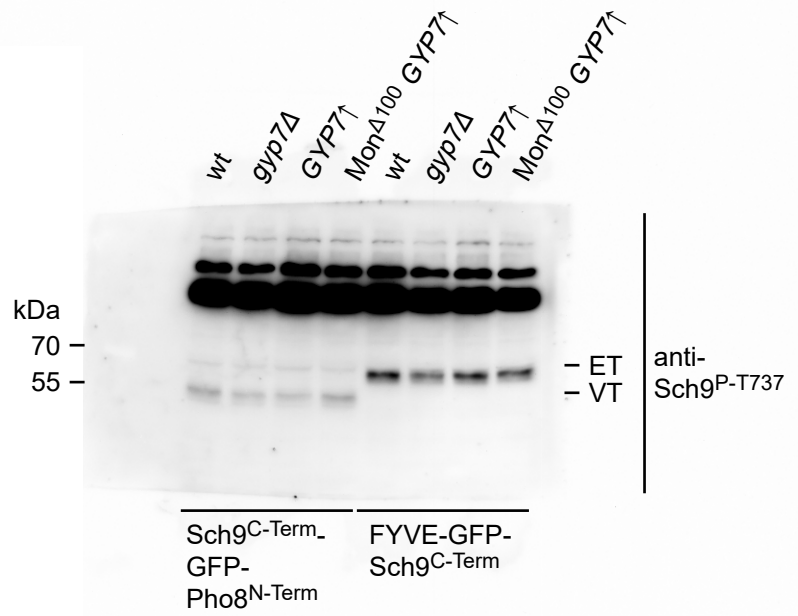

mNeon-Ypt7

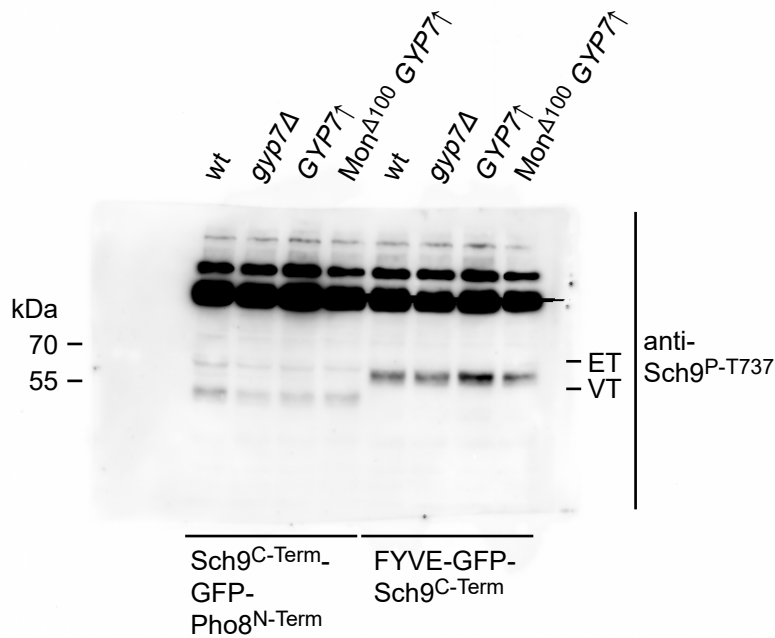

mNeon-Ypt7

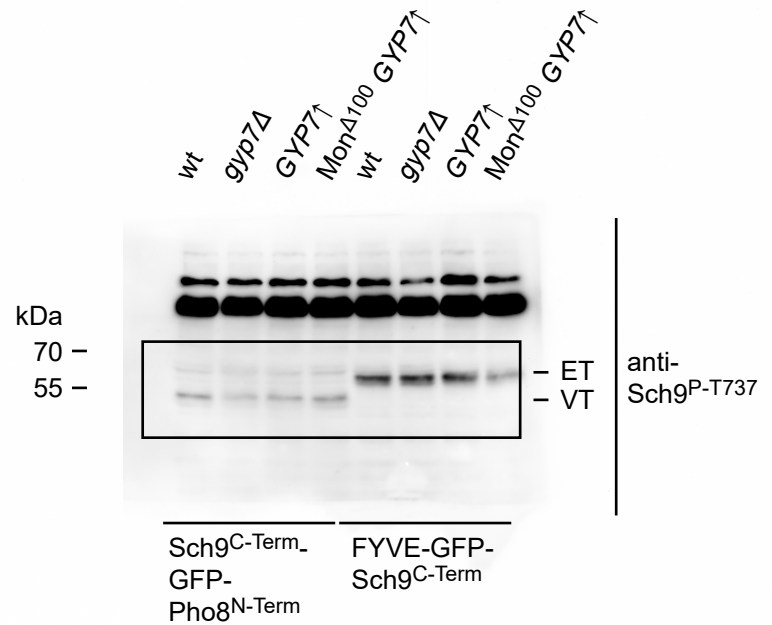

Supplement: SourceData FS5 — is the source file for Fig. S5. [file JCB_202305038_SourceDataFS5.pdf]
